# 5-[1-(Carb­oxy­meth­yl)pyridinium-4-yl]-1,2,3,4-tetra­zol-1-ide

**DOI:** 10.1107/S1600536810048403

**Published:** 2010-11-27

**Authors:** Li-ping Feng, Liang Zhao

**Affiliations:** a Department of Chemical & Environmental Engineering, Anyang Institute of Technology, Anyang 455000, People’s Republic of China

## Abstract

In the title compound, C_8_H_7_N_5_O_2_, the tetra­zole and pyridine rings are twisted from each other by a dihedral angle of 17.97 (1)°. The zwitterionic mol­ecules are connected by O—H⋯N hydrogen bonds into a chain parallel to [20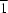]. Further C—H⋯O and C—H⋯N hydrogen bonds link the chains, building up a three-dimensional network.

## Related literature

For the chemisty of tetra­zoles and for related structures, see: Fu *et al.* (2009 ▶); Wen (2008 ▶); Dai & Fu (2008 ▶).
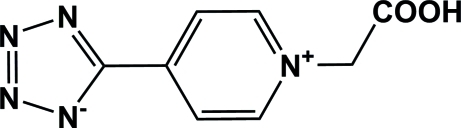


## Experimental

### 

#### Crystal data

C_8_H_7_N_5_O_2_
*M* *_r_* = 205.19
Monoclinic, 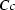

*a* = 8.8094 (18) Å
*b* = 9.3732 (19) Å
*c* = 11.189 (2) Å
β = 101.80 (3)°
*V* = 904.4 (3) Å^3^
*Z* = 4
Mo *K*α radiation
μ = 0.12 mm^−1^
*T* = 298 K
0.10 × 0.03 × 0.03 mm

#### Data collection

Rigaku Mercury2 diffractometer
Absorption correction: multi-scan (*CrystalClear*; Rigaku, 2005 ▶) *T*_min_ = 0.910, *T*_max_ = 1.000
4629 measured reflections
1043 independent reflections
971 reflections with *I* > 2σ(*I*)
*R*_int_ = 0.021

#### Refinement

*R*[*F* ^2^ > 2σ(*F* ^2^)] = 0.031
*wR*(*F* ^2^) = 0.082
*S* = 1.07
1043 reflections
137 parameters
2 restraints
H-atom parameters constrained
Δρ_max_ = 0.14 e Å^−3^
Δρ_min_ = −0.20 e Å^−3^

### 

Data collection: *CrystalClear* (Rigaku, 2005 ▶); cell refinement: *CrystalClear*; data reduction: *CrystalClear*; program(s) used to solve structure: *SHELXS97* (Sheldrick, 2008 ▶); program(s) used to refine structure: *SHELXL97* (Sheldrick, 2008 ▶); molecular graphics: *XP* in *SHELXTL* (Sheldrick, 2008 ▶) and *PLATON* (Spek, 2009 ▶); software used to prepare material for publication: *SHELXTL*.

## Supplementary Material

Crystal structure: contains datablocks I, global. DOI: 10.1107/S1600536810048403/dn2625sup1.cif

Structure factors: contains datablocks I. DOI: 10.1107/S1600536810048403/dn2625Isup2.hkl

Additional supplementary materials:  crystallographic information; 3D view; checkCIF report

## Figures and Tables

**Table 1:** Hydrogen-bond geometry (Å, °)

*D*—H⋯*A*	*D*—H	H⋯*A*	*D*⋯*A*	*D*—H⋯*A*
O1—H1*A*⋯N4^i^	0.82	1.84	2.648 (3)	170
C1—H1⋯O2^ii^	0.93	2.55	3.165 (3)	124
C1—H1⋯N3^iii^	0.93	2.59	3.440 (3)	152
C5—H5⋯N3^iv^	0.93	2.39	3.270 (3)	158

Symmetry codes: (i) 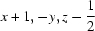; (ii) 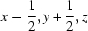; (iii) 
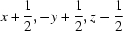
; (iv) 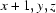.

**Fig. 1.**
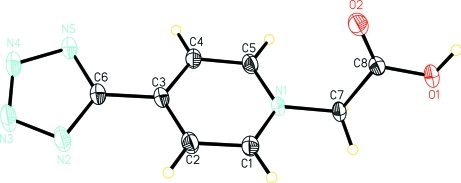
A view of the title compound with the atomic numbering scheme. Displacement ellipsoids were drawn at the 30% probability level. H atoms are represented as small spheres of arbitrary radii.

**Fig. 2.**
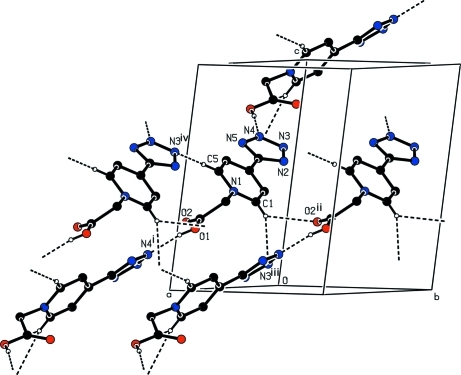
Partial packing view of the title compound showing the hydrogen bond pattern. H bonds are shown as dashed lines. H atoms not involved in hydrogen bonding have been omitted for clarity. [Symmetry codes: (i) x+1, -y, z-1/2; (ii) x-1/2, y+1/2, z; (iii) x+1/2, -y+1/2, z-1/2; (iv) x+1, y, z.]

## supplementary crystallographic information

### Comment

In the past few years, there was increasing interest in the chemistry of tetrazole derivatives owing their multiple coordination modes as ligands to metal ions and for the construction of novel metal-organic frameworks (Dai & Fu 2008; Fu *et al.*, 2009; Wen, 2008). We report here the crystal structure of the title compound, 5-(1-(carboxymethyl)pyridinium-4-yl)tetrazol-1-ide.

In the title compound (Fig.1), a carboxymethanide group was connected to the pyridine N atom, thus indicating a positive charge in the pyridine N atom. And the tetrazole ring was showing a negative charge to make the charge balance. The tetrazole and pyridine rings are twisted from each other by a dihedral angle of 17.97 (1)°. The geometric parameters of the tetrazole rings are comparable to those in related molecules (Fu *et al.*, 2009).

In the crystal structure, the zwitterionic molecules are connected by the O—H···N hydrogen bonds, with the O···N distance of 2.646 (2)Å. This H-bonds link the zwitterionic units into a one-dimentional chain parallel to the [2 0 -1] direction (Table 1 and Fig.2). Futhermore, C-H···O and C-H···N link the chain building up a three dimensionnal network (Table 1, Fig. 2).

### Experimental

5-(1-(carboxymethyl)pyridinium-4-yl)tetrazol-1-ide (4 mmol) was dissolved in ethanol (20 ml). The solution was allowed to evaporate to obtain colourless block-shaped crystals of the title compound.

### Refinement

All H atoms attached to C and atoms were fixed geometrically and treated as riding on their parent atoms with C–H = 0.93 Å (aromatic), 0.97 Å (methylene) and O-H = 0.82 Å with U*_iso_*(H) = 1.2*U_eq_*(C) or *U_iso_*(H) = 1.5*U_eq_*(O).

In the absence of significant anomalous scattering, the absolute configuration could not be reliably determined and then the Friedel pairs were merged and any references to the Flack parameter were removed.

### Crystal data

**Table:**

C_8_H_7_N_5_O_2_	*F*(000) = 424
*M**_r_* = 205.19	*D*_x_ = 1.507 Mg m^−^^3^
Monoclinic, *C**c*	Mo *K*α radiation, λ = 0.71073 Å
Hall symbol: C -2yc	Cell parameters from 2059 reflections
*a* = 8.8094 (18) Å	θ = 3.2–27.5°
*b* = 9.3732 (19) Å	µ = 0.12 mm^−^^1^
*c* = 11.189 (2) Å	*T* = 298 K
β = 101.80 (3)°	Block, colourless
*V* = 904.4 (3) Å^3^	0.10 × 0.03 × 0.03 mm
*Z* = 4	

### Data collection

**Table:**

Rigaku Mercury2 diffractometer	1043 independent reflections
Radiation source: fine-focus sealed tube	971 reflections with *I* > 2σ(*I*)
graphite	*R*_int_ = 0.021
Detector resolution: 13.6612 pixels mm^-1^	θ_max_ = 27.5°, θ_min_ = 3.2°
CCD profile fitting scans	*h* = −11→11
Absorption correction: multi-scan (*CrystalClear*; Rigaku, 2005)	*k* = −12→12
*T*_min_ = 0.910, *T*_max_ = 1.000	*l* = −14→14
4629 measured reflections	

### Refinement

**Table:**

Refinement on *F*^2^	Primary atom site location: structure-invariant direct methods
Least-squares matrix: full	Secondary atom site location: difference Fourier map
*R*[*F*^2^ > 2σ(*F*^2^)] = 0.031	Hydrogen site location: inferred from neighbouring sites
*wR*(*F*^2^) = 0.082	H-atom parameters constrained
*S* = 1.07	*w* = 1/[σ^2^(*F*_o_^2^) + (0.0478*P*)^2^ + 0.1896*P*] where *P* = (*F*_o_^2^ + 2*F*_c_^2^)/3
1043 reflections	(Δ/σ)_max_ < 0.001
137 parameters	Δρ_max_ = 0.14 e Å^−^^3^
2 restraints	Δρ_min_ = −0.20 e Å^−^^3^

### Special details

**Table:**

Geometry. All esds (except the esd in the dihedral angle between two l.s. planes) are estimated using the full covariance matrix. The cell esds are taken into account individually in the estimation of esds in distances, angles and torsion angles; correlations between esds in cell parameters are only used when they are defined by crystal symmetry. An approximate (isotropic) treatment of cell esds is used for estimating esds involving l.s. planes.
Refinement. Refinement of F^2^ against ALL reflections. The weighted R-factor wR and goodness of fit S are based on F^2^, conventional R-factors R are based on F, with F set to zero for negative F^2^. The threshold expression of F^2^ > 2sigma(F^2^) is used only for calculating R-factors(gt) etc. and is not relevant to the choice of reflections for refinement. R-factors based on F^2^ are statistically about twice as large as those based on F, and R- factors based on ALL data will be even larger.

### Fractional atomic coordinates and isotropic or equivalent isotropic displacement parameters (Å^2^)

**Table:**

	*x*	*y*	*z*	*U*_iso_*/*U*_eq_	
O1	1.1510 (2)	0.22028 (18)	0.28067 (17)	0.0424 (4)	
H1A	1.1988	0.1581	0.2523	0.064*	
O2	0.9872 (2)	0.04264 (18)	0.30239 (18)	0.0473 (4)	
N1	0.83175 (19)	0.21616 (19)	0.43834 (16)	0.0302 (4)	
N2	0.3137 (2)	0.1524 (3)	0.5601 (2)	0.0498 (6)	
N3	0.2244 (2)	0.0761 (3)	0.6185 (2)	0.0488 (6)	
N4	0.2990 (2)	−0.0370 (2)	0.66513 (19)	0.0411 (5)	
N5	0.4411 (2)	−0.0394 (2)	0.6403 (2)	0.0414 (5)	
C1	0.6906 (3)	0.2695 (3)	0.3907 (2)	0.0374 (5)	
H1	0.6795	0.3366	0.3283	0.045*	
C2	0.5627 (3)	0.2256 (3)	0.4332 (2)	0.0383 (5)	
H2	0.4658	0.2645	0.4011	0.046*	
C3	0.5786 (2)	0.1231 (2)	0.5242 (2)	0.0306 (4)	
C4	0.7249 (3)	0.0675 (2)	0.5702 (2)	0.0371 (5)	
H4	0.7380	−0.0023	0.6304	0.045*	
C5	0.8499 (3)	0.1156 (2)	0.5265 (2)	0.0375 (5)	
H5	0.9480	0.0788	0.5578	0.045*	
C6	0.4446 (2)	0.0779 (2)	0.57437 (19)	0.0320 (5)	
C7	0.9702 (2)	0.2699 (2)	0.3979 (2)	0.0339 (5)	
H7A	1.0494	0.2934	0.4690	0.041*	
H7B	0.9435	0.3567	0.3511	0.041*	
C8	1.0352 (2)	0.1622 (2)	0.32033 (19)	0.0308 (4)	

### Atomic displacement parameters (Å^2^)

**Table:**

	*U*^11^	*U*^22^	*U*^33^	*U*^12^	*U*^13^	*U*^23^
O1	0.0384 (8)	0.0479 (9)	0.0486 (9)	0.0017 (7)	0.0268 (8)	0.0014 (8)
O2	0.0452 (10)	0.0420 (9)	0.0610 (11)	−0.0036 (8)	0.0258 (9)	−0.0095 (8)
N1	0.0251 (9)	0.0346 (9)	0.0351 (9)	0.0004 (7)	0.0156 (7)	0.0006 (7)
N2	0.0292 (9)	0.0692 (14)	0.0553 (13)	0.0078 (10)	0.0184 (9)	0.0190 (11)
N3	0.0272 (9)	0.0736 (16)	0.0495 (11)	−0.0015 (10)	0.0168 (8)	0.0095 (11)
N4	0.0321 (9)	0.0537 (12)	0.0429 (10)	−0.0090 (9)	0.0202 (8)	−0.0053 (9)
N5	0.0333 (10)	0.0468 (11)	0.0504 (11)	−0.0008 (8)	0.0232 (9)	0.0039 (9)
C1	0.0328 (11)	0.0421 (12)	0.0406 (11)	0.0048 (9)	0.0152 (9)	0.0104 (10)
C2	0.0256 (10)	0.0484 (13)	0.0431 (13)	0.0047 (9)	0.0121 (9)	0.0081 (10)
C3	0.0247 (9)	0.0385 (11)	0.0312 (10)	−0.0004 (8)	0.0116 (8)	−0.0031 (8)
C4	0.0295 (10)	0.0417 (11)	0.0434 (12)	0.0028 (9)	0.0150 (9)	0.0125 (11)
C5	0.0246 (9)	0.0462 (12)	0.0438 (12)	0.0072 (9)	0.0119 (9)	0.0103 (10)
C6	0.0276 (10)	0.0389 (11)	0.0320 (10)	−0.0029 (8)	0.0117 (9)	−0.0016 (8)
C7	0.0296 (11)	0.0359 (11)	0.0419 (12)	−0.0011 (8)	0.0205 (9)	0.0005 (9)
C8	0.0284 (9)	0.0334 (10)	0.0331 (10)	0.0035 (8)	0.0120 (8)	0.0014 (9)

### Geometric parameters (Å, °)

**Table:**

O1—C8	1.311 (3)	C1—H1	0.9300
O1—H1A	0.8200	C2—C3	1.386 (3)
O2—C8	1.200 (3)	C2—H2	0.9300
N1—C1	1.345 (3)	C3—C4	1.388 (3)
N1—C5	1.350 (3)	C3—C6	1.469 (3)
N1—C7	1.474 (3)	C4—C5	1.370 (3)
N2—N3	1.329 (3)	C4—H4	0.9300
N2—C6	1.330 (3)	C5—H5	0.9300
N3—N4	1.299 (3)	C7—C8	1.517 (3)
N4—N5	1.337 (3)	C7—H7A	0.9700
N5—C6	1.328 (3)	C7—H7B	0.9700
C1—C2	1.373 (3)		
			
C8—O1—H1A	109.5	C5—C4—H4	120.1
C1—N1—C5	120.71 (18)	C3—C4—H4	120.1
C1—N1—C7	120.49 (18)	N1—C5—C4	120.5 (2)
C5—N1—C7	118.77 (18)	N1—C5—H5	119.8
N3—N2—C6	104.1 (2)	C4—C5—H5	119.8
N4—N3—N2	109.6 (2)	N5—C6—N2	112.5 (2)
N3—N4—N5	110.4 (2)	N5—C6—C3	124.28 (19)
C6—N5—N4	103.3 (2)	N2—C6—C3	123.2 (2)
N1—C1—C2	120.6 (2)	N1—C7—C8	112.40 (17)
N1—C1—H1	119.7	N1—C7—H7A	109.1
C2—C1—H1	119.7	C8—C7—H7A	109.1
C1—C2—C3	119.7 (2)	N1—C7—H7B	109.1
C1—C2—H2	120.2	C8—C7—H7B	109.1
C3—C2—H2	120.2	H7A—C7—H7B	107.9
C2—C3—C4	118.6 (2)	O2—C8—O1	127.1 (2)
C2—C3—C6	120.81 (19)	O2—C8—C7	123.7 (2)
C4—C3—C6	120.52 (19)	O1—C8—C7	109.16 (17)
C5—C4—C3	119.8 (2)		
			
C6—N2—N3—N4	0.3 (3)	N4—N5—C6—N2	1.3 (3)
N2—N3—N4—N5	0.5 (3)	N4—N5—C6—C3	−179.9 (2)
N3—N4—N5—C6	−1.0 (3)	N3—N2—C6—N5	−1.0 (3)
C5—N1—C1—C2	1.9 (4)	N3—N2—C6—C3	−179.9 (2)
C7—N1—C1—C2	−176.2 (2)	C2—C3—C6—N5	164.3 (2)
N1—C1—C2—C3	−1.5 (4)	C4—C3—C6—N5	−17.9 (3)
C1—C2—C3—C4	0.1 (3)	C2—C3—C6—N2	−17.0 (3)
C1—C2—C3—C6	177.9 (2)	C4—C3—C6—N2	160.8 (2)
C2—C3—C4—C5	0.9 (4)	C1—N1—C7—C8	−108.5 (2)
C6—C3—C4—C5	−176.9 (2)	C5—N1—C7—C8	73.4 (3)
C1—N1—C5—C4	−0.9 (4)	N1—C7—C8—O2	−5.8 (3)
C7—N1—C5—C4	177.2 (2)	N1—C7—C8—O1	175.68 (18)
C3—C4—C5—N1	−0.5 (4)		

### Hydrogen-bond geometry (Å, °)

**Table:**

*D*—H···*A*	*D*—H	H···*A*	*D*···*A*	*D*—H···*A*
O1—H1A···N4^i^	0.82	1.84	2.648 (3)	170
C1—H1···O2^ii^	0.93	2.55	3.165 (3)	124
C1—H1···N3^iii^	0.93	2.59	3.440 (3)	152
C5—H5···N3^iv^	0.93	2.39	3.270 (3)	158

Symmetry codes: (i) *x*+1, −*y*, *z*−1/2; (ii) *x*−1/2, *y*+1/2, *z*; (iii) *x*+1/2, −*y*+1/2, *z*−1/2; (iv) *x*+1, *y*, *z*.
